# Rheological Properties and Age-Related Changes of the Human Vitreous Humor

**DOI:** 10.3389/fbioe.2018.00199

**Published:** 2018-12-18

**Authors:** Nguyen K. Tram, Katelyn E. Swindle-Reilly

**Affiliations:** ^1^Department of Biomedical Engineering, The Ohio State University, Columbus, OH, United States; ^2^William G. Lowrie Department of Chemical and Biomolecular Engineering, The Ohio State University, Columbus, OH, United States; ^3^Department of Ophthalmology and Visual Science, The Ohio State University, Columbus, OH, United States

**Keywords:** rheometry, vitreous, aging, ocular biomechanics, liquefaction, eye, viscoelasticity, floaters

## Abstract

The vitreous humor is a fragile, transparent hydrogel situated between the lens and the retina, occupying 80% of the eye's volume. Due to its viscoelastic behavior, the vitreous serves as a mechanical damper for the eye, absorbing impacts, and protecting the lens and retina. The vitreous liquefies with age, which compromises its function as a shock absorber and causes complications including retinal detachment, macular holes, and vitreous hemorrhage. Studies on the viscoelastic properties of the vitreous have been limited. Rheological testing of the vitreous has commonly been done on non-primate mammalian species. Human vitreous rheological properties have been previously reported; however, various measurement techniques were used, resulting in data that differed by orders of magnitude. Shear rheometry is commonly used to characterize soft tissues and hydrogels such as the vitreous humor. However, no human vitreous rheological data have been reported using this technique, preventing direct comparison to other published work. Additionally, no age-related changes in the mechanical properties of the human vitreous humor have been reported. Human vitreous samples (*n* = 39, aged 62 ± 15 years) were tested using a shear rheometer. Small amplitude oscillatory shear and creep experiments were performed. The linear viscoelastic region of the human vitreous was found to be below 1% strain. The solid phase of the old human vitreous was found to be stiffer than the young human vitreous and the porcine vitreous. The stiffness of the human vitreous gel also appeared to be positively correlated with age. Vitreous dehydration due to a decrease in hyaluronic acid concentration with age was proposed to cause the stiffening of the solid phase of the vitreous gel. Vitreous liquefaction, therefore, might be characterized as a simultaneous increase in liquid volume and localized stiffening of the vitreous gel. The phase separation of the vitreous humor with age has been hypothesized as the cause of many vitreous-related complications. This study provides viscoelastic properties and age-related changes of the human vitreous humor, which will aid in the design of biomimetic vitreous substitutes, enhancement in analyzing intravitreal transport of therapeutics, and understanding the pathological conditions of the vitreous humor.

## Introduction

The vitreous humor is a fragile, transparent hydrogel situated between the lens and the retina, occupying 80% of the eye's volume. The vitreous is a viscoelastic gel composed of ~99% water with a framework of type II collagen and hyaluronic acid. The hyaluronic acid coils are interspersed in the network of collagen type II and collagen type IX (Le Goff, 2008). The vitreous humor serves as a mechanical damper for the eye, absorbing impacts and protecting the lens and retina. The vitreous also participates in the growth processes of the eye during development (Coulombre, 1956). However, the vitreous liquefies with age due to a variety of factors such as oxidative damage, digestion by enzymes, and collagen mutations (Okada et al., 1989; Brown et al., 1996), resulting in phase-separation and gel network collapse. Vitreous liquefaction compromises the vitreous' function as a shock absorber and causes complications including rhegmatogenous retinal detachment, macular holes, vitreous hemorrhage, and vitreous floaters (Los et al., 2003). It is important to study the mechanical properties of the vitreous to gain insight into diseases that might be caused by vitreous liquefaction, to study the release and transport profile of intraocular drugs injected into the vitreous humor, and to guide the design of vitreous substitutes that can replace both the form and function of the natural vitreous humor. However, quantitative measurements of human vitreous humor, especially its rheological properties, are scarce in the literature.

A number of studies have quantified the mechanical properties of the mammalian vitreous using bulk measurements (Bettelheim and Wang, 1976; Weber et al., 1982; Tokita et al., 1984), magnetic microrheology (Lee et al., 1992, 1994; Watts et al., 2014), *in vivo* visual tracking (Zimmerman, 1980), acoustic and magnetic resonance imaging (MRI) techniques (Walton et al., 2002; Stein et al., 2018), and shear rheometry (Nickerson et al., 2005, 2008; Suri and Banerjee, 2006; Swindle et al., 2008; Swindle-Reilly et al., 2009, 2016; Sharif-Kashani et al., 2011; Filas et al., 2014; Colter et al., 2015; Silva et al., 2017; Shafaie et al., 2018). In recent years, shear rheometry has become a common technique for testing the vitreous humor, since this technique can report various rheological properties pertaining to the viscoelasticity of the vitreous gel. In particular, the rheological parameters of interest are storage modulus and loss modulus, which describe the solid and viscous qualities of a material, respectively. Due to the scarcity of human tissues, only a handful of studies reported human vitreous mechanical data. Previous data on the vitreous humor have been commonly reported on porcine, bovine, ovine, or rabbit eyes.

While the vitreous is well-known to liquefy with age, age-related changes in the mechanical properties of the vitreous remain elusive. Colter et al. (2015) is the only paper to date that investigated age-related changes in the mechanical data of the vitreous humor, using ovine eyes. The authors claimed that the storage and loss moduli of the adult ovine vitreous were lower than those of the infant vitreous and higher than those of the premature vitreous. However, there were no significant differences between the ages tested. Additionally, the vitreous was tested whole and assumed to be homogeneous. Since the hallmark of vitreous liquefaction is phase separation of the vitreous humor, which results in pockets of liquid inside the vitreous, a different approach to mechanically test the vitreous could be used to capture its heterogeneity. In this study, both the solid and liquid phases of the vitreous were mechanically tested and correlated to changes with age.

This study utilizes a technique common in characterizing the material properties of hydrogels, shear rheometry, allowing for a broader comparison of our results to previous studies. This study also fills in this scientific gap by using this technique to quantitatively measure human vitreous rheological properties, with an emphasis on the differences between vitreous samples from young and old tissue samples on both the solid and liquid phases of the vitreous.

## Materials and Methods

### Post-mortem and Post-dissection Time Studies

To study the effect of post-mortem and post-dissection time on the viscoelastic properties of the vitreous, fresh porcine eyes (*n* = 15, 1 unpaired eye, 14 paired eyes) were obtained from a local abattoir (Delaware Meats, Delaware, Ohio). The sclera was cut along the nasotemporal meridian around the posterior end (avoiding the cornea). Blunted surgical forceps were used to remove the vitreous through the corneoscleral shell, and the vitreous was immediately placed on ice. The vitreous was extracted from the porcine eye within 2 h post mortem and tested immediately, after 2 h, or after 4 h (same sample) to determine the effect of storing dissected vitreous on its material properties. A number of intact eyes were reserved, dissected, and tested 24 or 48 h later, to determine the effect of post-mortem time on the material properties of the vitreous. Intact eyes and dissected vitreous samples were stored in sealed containers at 4°C.

### Rheology of Human Vitreous Humor

Human vitreous tissues were obtained from other research groups utilizing human eye samples (aged 62 ± 15 years old, *n* = 39, 3 unpaired eyes, 36 paired eyes). Human eyes were provided by the Lions Eye Bank of West Central Ohio (Dayton, Ohio) and the Central Ohio Lions Eye Bank (Columbus, Ohio). The intact eyes were submerged in saline in vials and stored at 4°C prior to testing. Vitreous samples were collected (using the same aforementioned technique for porcine eyes) within 48 h post mortem and tested within 4 h after dissection.

**Figure 1:**
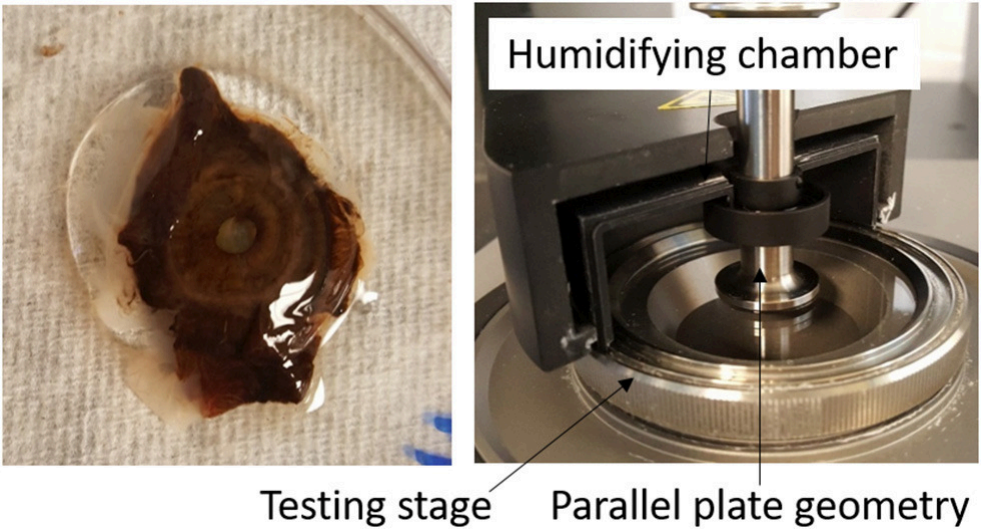
**(Left)** Intact human vitreous humor sample with the lens, ciliary body, and iris tissues attached. **(Right)** Experimental set-up. The vitreous sample is sandwiched between the parallel plate geometry and testing stage. A humidifying chamber filled with phosphate buffered saline was used to prevent dehydration of the vitreous sample.

Approximately 0.3 ml of the vitreous, free of other ocular tissues, was cut using forceps and surgical scissors and placed onto the quartz testing stage of a Kinexus ultra+ rheometer (Malvern Instruments Ltd, Worcestershire, UK). A 20-mm parallel plate geometry was lowered onto the vitreous sample to a working gap of 1 mm, which was determined to provide good contact between the geometry and the vitreous sample without damaging the vitreous (zero normal force). To prevent the slippage effect, 600-grit sandpaper was applied to the test geometry of the rheometer (Yoshimura and Prud'homme, 1988; Sharif-Kashani et al., 2011; Silva et al., 2017). The testing stage was set to 37°C and a humidifying chamber filled with phosphate buffered saline was attached around the geometry and testing stage to simulate *in vivo* conditions and minimize sample dehydration (Figure 1). The liquid portion of the vitreous humor sample was also tested using the parallel plate geometry with a 0.1 mm gap. The solid phase of the vitreous was the cohesive portion of the tissue that could be picked up with forceps, while the liquid phase could not be picked up with forceps and was transferred to the testing stage using a dropper.

First, amplitude sweep tests were conducted on the solid phase of the vitreous at frequencies of 0.1 and 1 Hz and amplitudes ranging from 0.1 to 1,000%. Thereafter, frequency sweep tests with strain amplitude of 0.5% (found to be within the linear viscoelastic region) were conducted with frequency ranges from 0.01 to 100 Hz. It was found that once the frequency passes 1 Hz, the inertia effect of the geometry dominates the response of the vitreous sample, resulting in an artificial increase in modulus of the vitreous, which has been previously reported (Nickerson et al., 2005, 2008; Sharif-Kashani et al., 2011; Filas et al., 2014; Colter et al., 2015; Shafaie et al., 2018). Therefore, only data from frequencies below 1 Hz were reported.

Creep tests were conducted at constant shear stress of 1 Pa for 1,000 s to cover the different viscoelastic responses as described in (Sharif-Kashani et al., 2011). Compliance was calculated as strain over stress and fitted to a viscoelastic spectra model with two Voigt-Kelvin elements and a dashpot in series as described in Sharif-Kashani et al. (2011). The mathematical expression of this model is

(1) $$\begin{array}{l} J\left(t\right)=\;\underset{k}{\sum }J_{k}\left(1-e^{-\left(\frac{t}{t_{k}}\right)}\right)+\;\frac{t}{\eta_{m}} \end{array}$$

where *k* = 2, which is the number of elements, J_k_ is the compliance of each element, t_k_ is the retardation time, and η_m_ is the viscosity representing the viscous behavior of the vitreous at steady state. The variables J_k_, t_k_, and η_m_ were determined by fitting the compliance-time curve to the equation above using MATLAB (The MathWorks, Inc., Natick, MA).

### Statistical Analysis

In a few cases, experimental or sample artifacts gave values that were distinctly different from the majority of the sample values. In these cases, the outliers were eliminated as previously described (Havilcek and Crain, 1988; Lee et al., 1992). Briefly, the mean and standard deviation of each variable were calculated. Values > 2.5 standard deviations from the mean were considered outliers, and new mean and standard deviation were calculated. This process was repeated until all data points were within the 2.5 standard deviations of the mean. This procedure removed no more than two data points per variable, except for one case where 4 outliers were removed (Supplemental figure). It was determined that the presence or absence of these outliers does not affect the results presented, therefore the outliers were eliminated. Statistical analyses were implemented with Minitab software (version 18.1; Minitab, Inc., State College, PA). Spearman correlations were used to determine the correlation between rheological properties and age. The difference between young and old samples was set at 65 years of age (old age as defined by the American Heart Association). A two-tailed Student's *t*-test was used to compare rheological data from the liquid component of the young and old human vitreous. One-way ANOVA, with *post-hoc* pairwise comparison using Tukey test, was used to compare the rheological data of the solid component of human vitreous samples from both age groups and porcine vitreous samples. ANOVA was also used to compare the rheological results of porcine eyes from the post-mortem and post-dissection time studies. The null hypotheses stated that there is no difference between the groups for each test. An alpha value of 0.05 was used for statistical significance.

## Results

**Figure 2:**
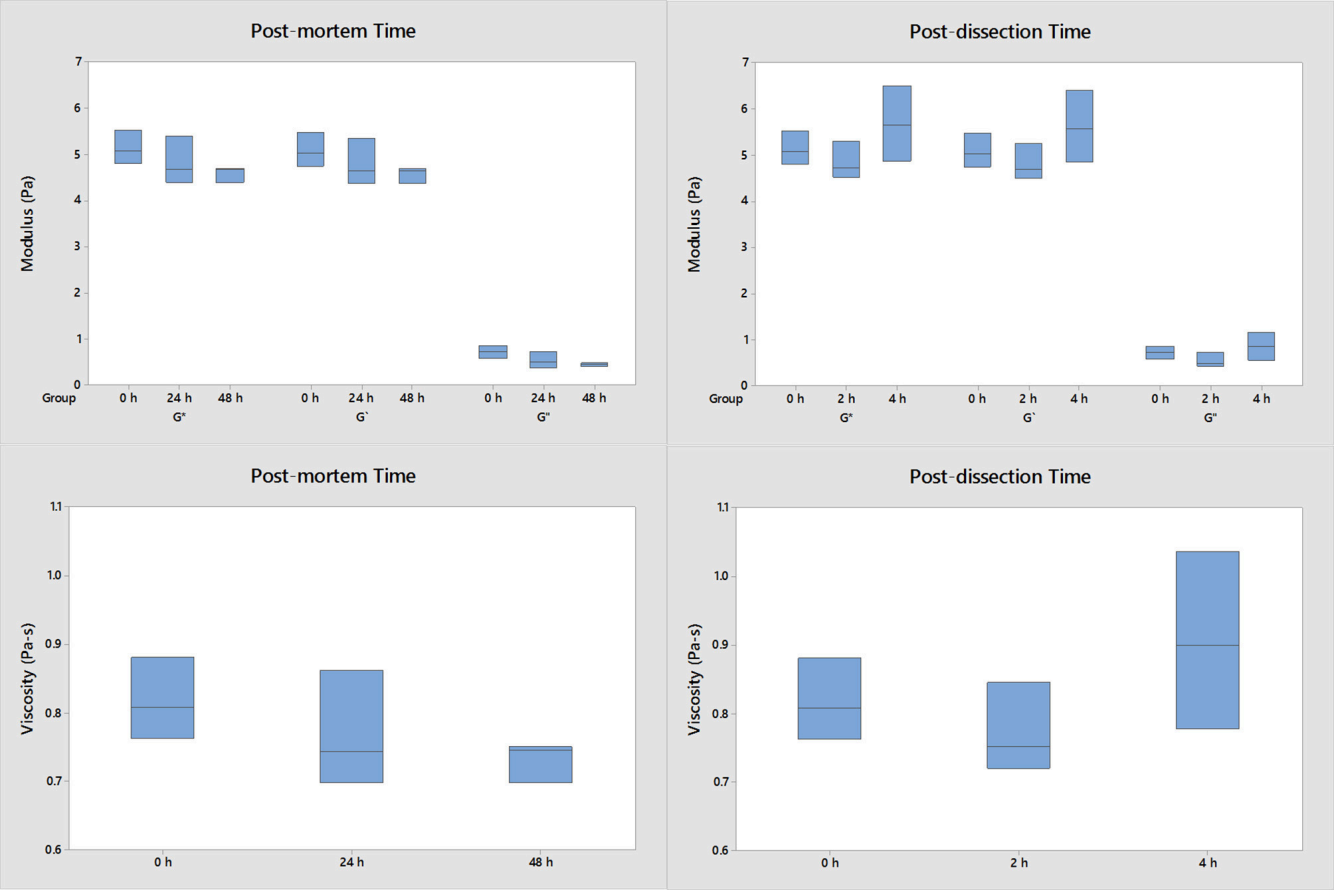
The effect of post-dissection time and post-mortem time on the moduli and viscosity of porcine vitreous (*n* = 3 for each group). The data were collected at 1 Hz and 0.5% strain. Both the intact eyes and dissected vitreous samples were stored at 4°C. No significant differences were detected when the vitreous samples were harvested within 48 h post-mortem and used within 4 h after extraction compared to fresh samples.

Post-mortem time and post-dissection time do not have an effect on the mechanical properties of the porcine vitreous (Figure 2). No statistically significant differences were detected in these studies. The porcine moduli and viscosity did not significantly change if tested within 48 h post-mortem and within 4 h after extraction compared to fresh samples. All human samples were tested within 48 h post-mortem and 4 h of extraction.

**Figure 3:**
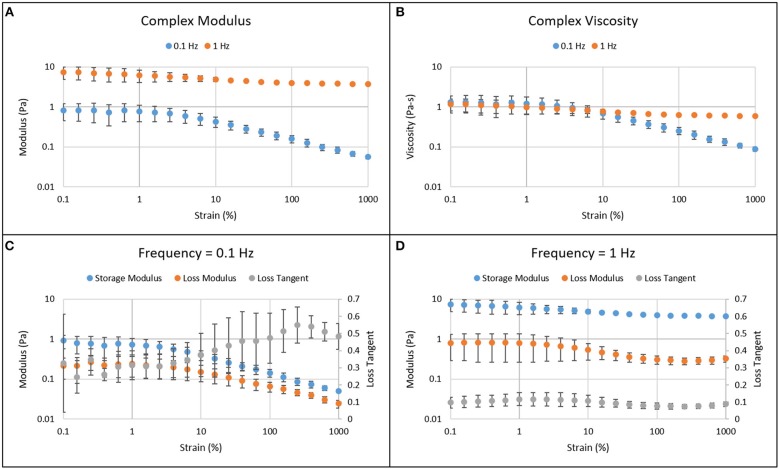
Amplitude sweep results of the solid phase of human vitreous samples at frequencies of 0.1 and 1 Hz. The complex modulus **(A)** and viscosity **(B)** are larger when the amplitude sweep experiment was done at a higher frequency. Below 1% strain, the moduli and viscosity are independent of strain, which is the linear viscoelastic region for both low **(C)** and high **(D)** frequencies. As strain increases above 1%, the moduli and viscosity decrease while loss tangent increases. The error bar represents 95% confidence interval.

Figure 3 shows the results from amplitude sweep experiments. The complex, storage, and loss moduli and the viscosity were larger at higher frequency (1 Hz) compared to those at lower frequency (0.1 Hz). The linear viscoelastic region for human vitreous was determined to be below 1% strain. The moduli and viscosity are independent of strain below 1%. Above 1% strain, the moduli and viscosity decrease while loss tangent increases as seen at 0.1 Hz frequency. As a result, 0.5% strain was used in further testing. The moduli, viscosity, and loss tangent appeared to be less dependent on strain amplitude at 1 Hz frequency.

**Figure 4:**
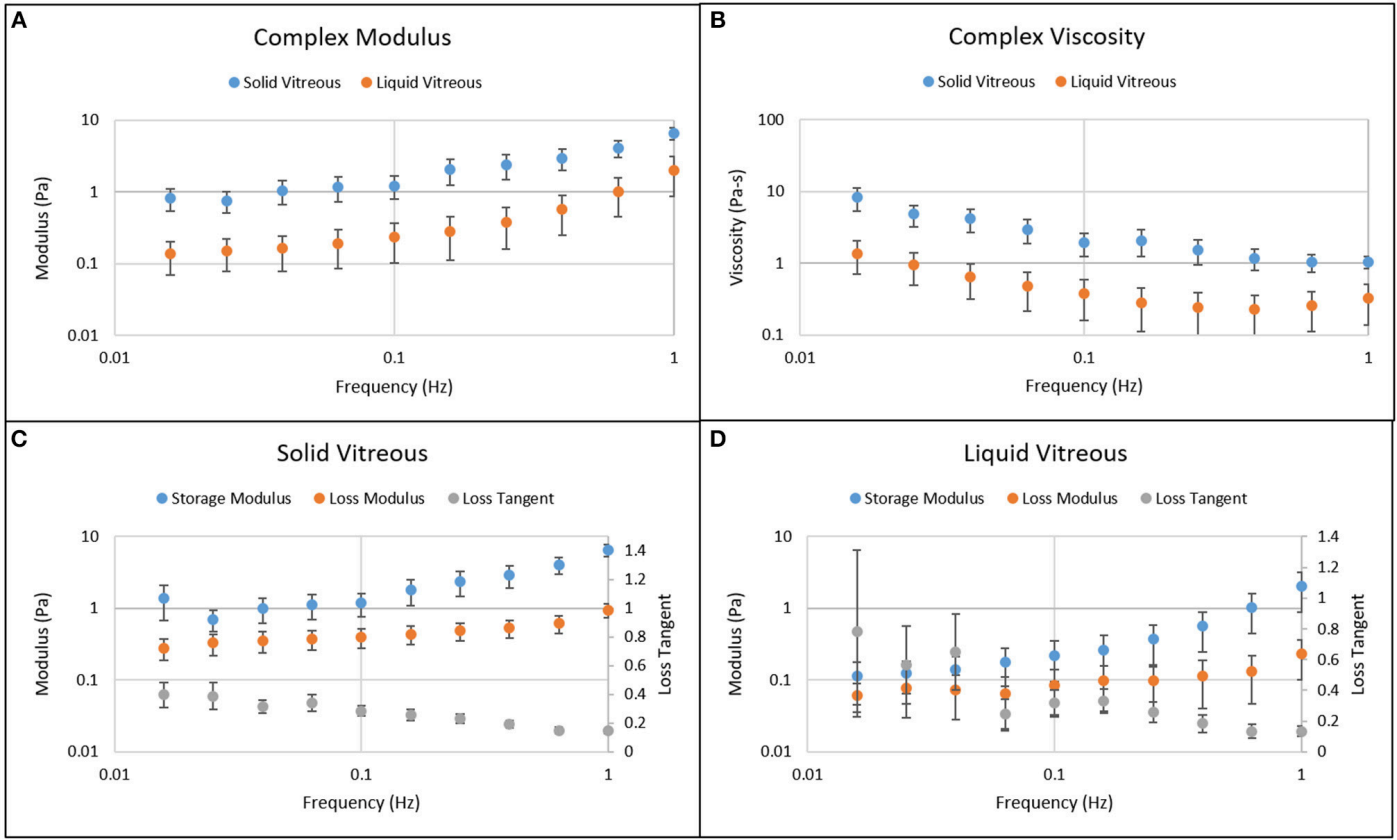
Frequency sweep results of the solid and liquid phases of the human vitreous samples at 0.5% strain. **(A)** Complex modulus of solid and liquid phases of vitreous. **(B)** Complex viscosity of solid and liquid phases of vitreous. **(C)** Moduli and loss tangent of solid vitreous. **(D)** Moduli and loss tangent of liquid vitreous. The moduli and viscosity of the solid vitreous are higher than those of the liquid vitreous. As the frequency increases, the moduli increase while the viscosity and loss tangent decrease, suggesting that the vitreous behaves more as a solid body with increasing frequency. The error bar represents 95% confidence interval.

Figure 4 shows the results from frequency sweep experiments. The moduli and viscosity of the solid phase of the vitreous were higher than those of the liquid part of the vitreous (${\text{G}}_{\text{solid}}^{*}$ = 0.83 ± 0.14 Pa > ${\text{G}}_{\text{liquid}}^{*}$ = 0.14 ± 0.12 Pa, *p* < 0.0001, measured at 0.02 Hz and 0.5% strain). As the frequency increased, the moduli increased while the loss tangent decreased, suggesting that the vitreous behaves more as a solid body with increasing frequency. The complex modulus ranged from 1 to 7 Pa for the solid phase of the vitreous and from 0.1 to 2 Pa for the liquid part of the vitreous. The complex viscosity ranged from 1 to 10 Pa-s for the solid phase and from 0.2 to 1 Pa-s for the liquid phase. The loss tangent ranged from 0.1 to 0.4 for the solid phase and from 0.1 to 0.7 for the liquid phase. No significant differences were found between the right and left eyes (9 OD vs. 9 OS, 2-sample *t*-test, *p* = 0.720), between male and female eyes (4 female vs. 19 male, 2-sample *t*-test, *p* = 0.654), and between normal eyes and eyes with intraocular lens (20 normal vs. 3 with intraocular lens, 2-sample *t*-test, *p* = 0.443).

**Figure 5:**
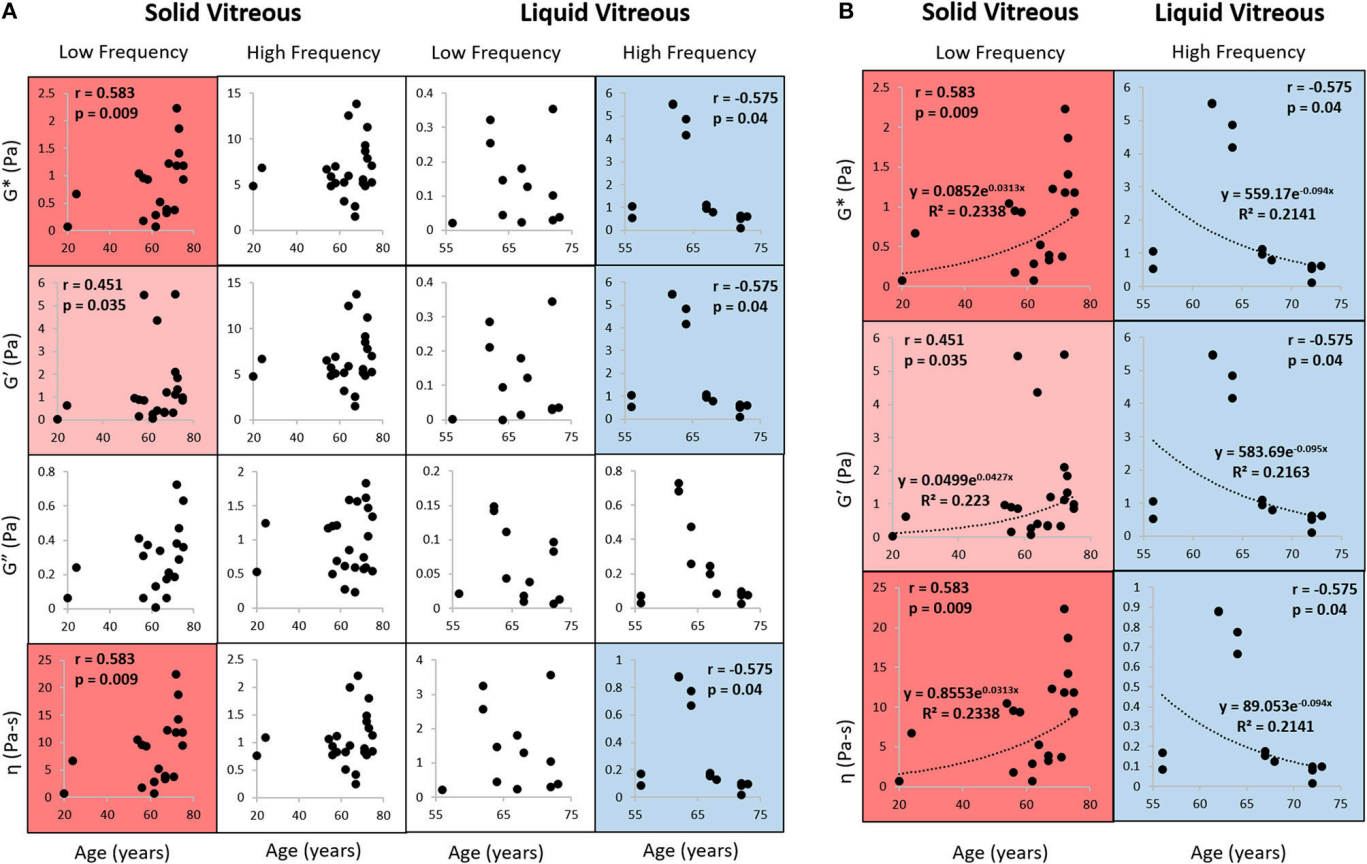
Rheological properties correlation analysis. **(A)** Complex modulus, storage modulus, loss modulus, and complex viscosity were compared against age using Spearman correlation analysis. **Left:** y-axis variables (complex viscosity, loss modulus, storage modulus, complex modulus); **Bottom:** x-axis variable (age); **Top:** sample phase (solid vs. liquid) and frequency. Data were obtained at 0.5% strain and at low frequency (0.02 Hz) or high frequency (1 Hz). The movement of the geometry at high frequencies can cause an inertial effect that artificially increases the measured modulus of the vitreous, which has been shown in previous studies, so only data from frequencies below 1 Hz are shown. Numeric correlation coefficients shown for positive (red) and negative (blue) correlations (light shading indicates *p* < 0.05; dark shading indicates *p* < 0.01). White boxes indicate no significant correlation (*p* > 0.05). The parameters from solid vitreous are positively correlated with age at low frequency, while those from liquid vitreous are negatively correlated with age at high frequency. Loss modulus did not correlate with age. **(B)** Exponential fit to data with significant Spearman correlations. G*, complex modulus; G′, storage modulus; G″, loss modulus; η, viscosity.

Figure 5 shows the Spearman correlation analysis of human vitreous rheological properties as a function of age. The Spearman correlation coefficient (rho) varies between +1 and −1. A rho of +1 indicates a perfect correlation between the factors and a rho of −1 indicates a perfect negative correlation between the factors. The closer rho is to zero, the weaker the correlation between the factors. This figure looks at the effects of age at high and low frequencies on the properties of the vitreous humor. Complex modulus, storage modulus, and complex viscosity were found to positively correlate with age at low frequency (0.02 Hz) for the solid vitreous (red boxes) and negatively correlate with age at high frequency (1 Hz) for the liquid vitreous (blue boxes). White boxes represent no significant correlation. In this study, loss modulus did not correlate with age.

**Figure 6:**
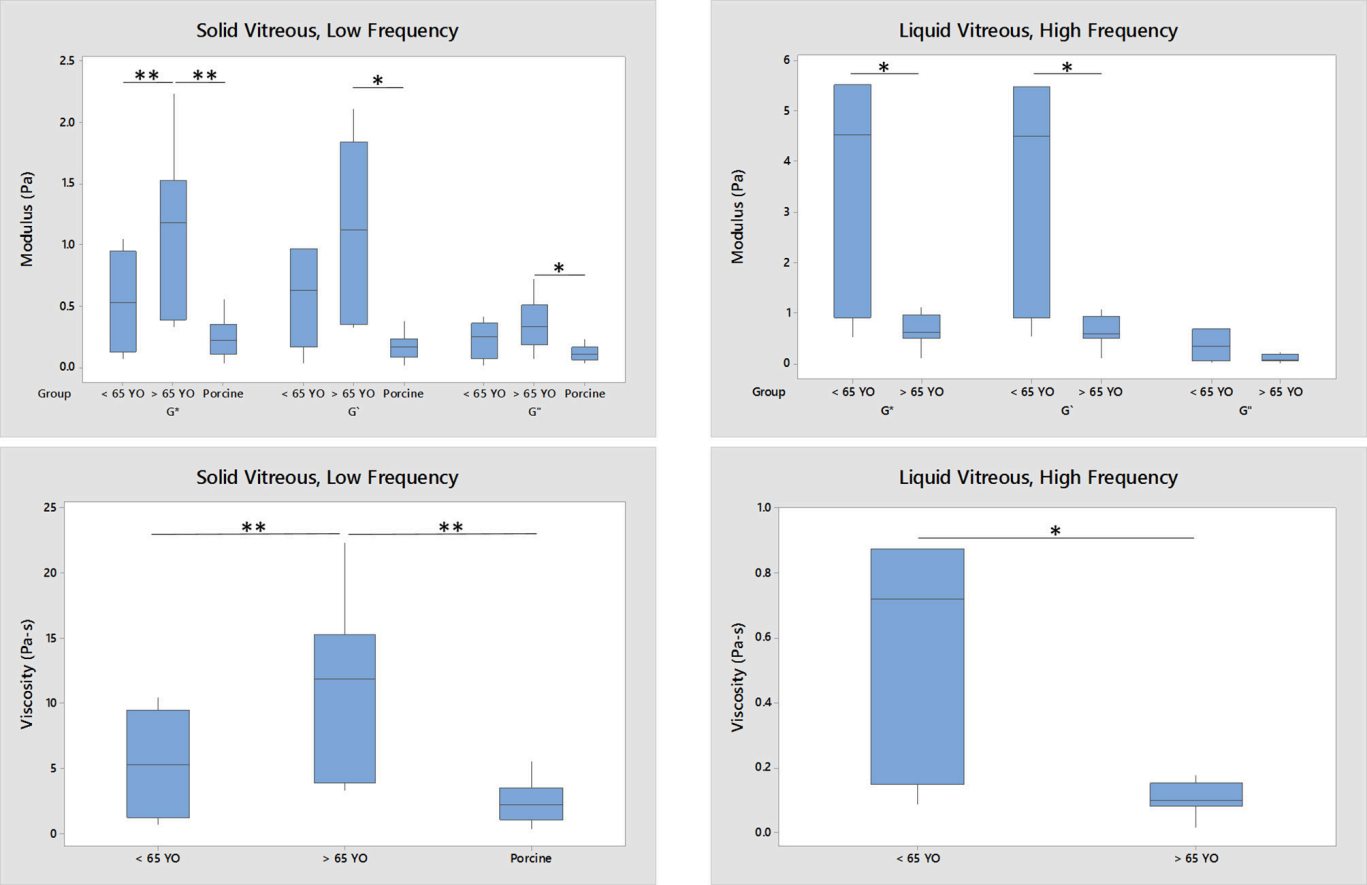
Age-related difference between young (*n* = 11 for solid, *n* = 6 for liquid), old (*n* = 12 for solid, *n* = 7 for liquid) human vitreous samples and porcine (*n* = 15, only solid) vitreous samples (*p < 0.05; **p < 0.0001). The data were collected at 0.1 Hz and 0.5% strain. For the solid phase of the vitreous, the moduli and viscosity of the old human vitreous are significantly larger than those of the young human vitreous and porcine vitreous. For the liquid phase of the vitreous, the moduli and viscosity of the old group are significantly lower than those from the young group. No liquid phase of porcine vitreous was tested.

Figure 6 further analyzes the correlations shown in Figure 5. For the solid component of the vitreous at low frequency, the rheological data from old human vitreous samples (age > 65 years, *n* = 12) were significantly higher than those from the young human vitreous samples (age < 65 years, *n* = 11) and the porcine vitreous samples (average age 6 months, *n* = 15). For the liquid component of the vitreous at high frequency, the rheological results from the old human samples were significantly smaller than those from the young human samples. Due to the absence of liquid separation in the porcine vitreous samples, the liquid portion of the vitreous could not be tested.

**Figure 7:**
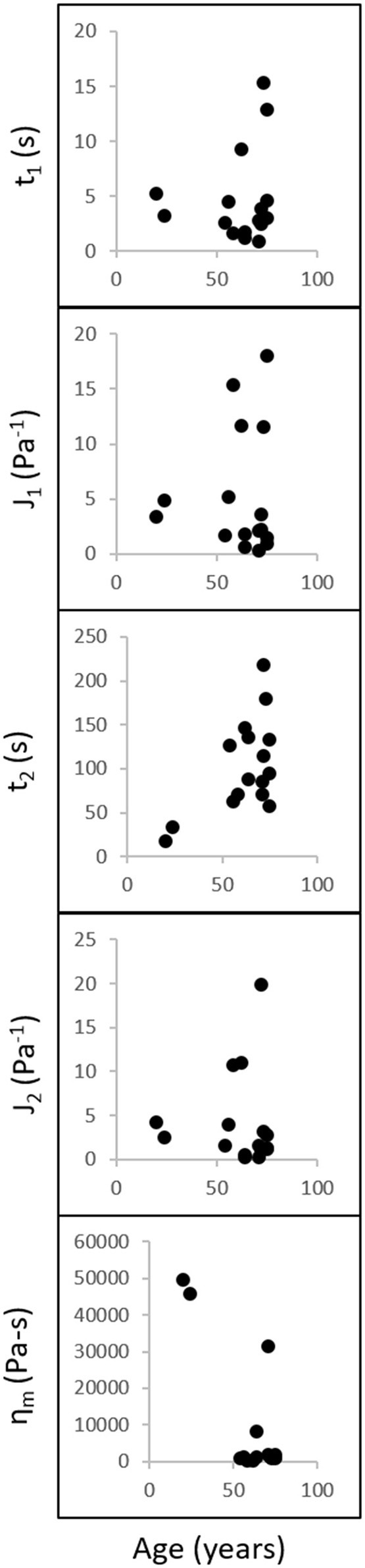
Discrete retardation parameters correlation analysis. Retardation times, compliances, and viscosity (y-axis) were compared against age (x-axis) using Spearman correlation analysis. **Left:** y-axis variables; **Bottom:** x-axis variable (age). Data were obtained at a constant shear stress of 1 Pa for 1,000 s. No correlations were found between the discrete retardation parameters and age.

Figure 7 shows the discrete retardation parameters correlation results from creep testing. No significant correlation was found between the discrete retardation parameters and age. Table 1 shows the parameters calculated from the discrete retardation model for creep test. The calculated first retardation time and first compliance are significantly different than those reported in Sharif-Kashani et al. (2011). It should be noted that this paper used human vitreous samples, as opposed to porcine vitreous sample used in Sharif-Kashani et al. (2011).

**Table 1:** Parameters of discrete retardation model for creep test.

	**Species**	**t_**1**_ (s)**	**J_**1**_ (Pa^−1^)**	**t_**2**_ (s)**	**J_**2**_ (Pa^−1^)**	**η_m_ (Pa^−s^)**
This study	Human (*n* = 16)	4.70 ± 1.05	5.28 ± 1.41	102.03 ± 13.23	4.08 ± 1.34	9205.31 ± 4222.72
(Sharif-Kashani et al., 2011)	Porcine (*n* = 9)	1.97 ± 0.69	0.83 ± 0.05	90.00 ± 30.83	1.30 ± 0.83	1057.0 ± 407.3
*p*-value^*^		0.022	0.007	0.480	0.059	0.073

* *Two-sample t-test comparing values from this paper vs. reported in Sharif-Kashani et al. (2011)*.

## Discussion

This is the first study that extensively reports the rheological properties and age-related changes of the human vitreous using shear rheometry, which is a commonly used rheological technique for soft tissues and hydrogels. Previous rheological studies on the vitreous used non-human species such as porcine, bovine, ovine, rabbits, and goats. Weber et al. (1982), Zimmerman (1980), Lee et al. (1992), and Shafaie et al. (2018) were the only papers that reported human vitreous rheological data, but each used different techniques (Table 2). Lee et al. (1992) performed creep experiments on magnetic microspheres embedded in the vitreous and converted the measured compliance to dynamic moduli. The age of the human samples was not reported, and it was claimed that the central porcine vitreous closely resembled that of the human vitreous, although the pig moduli appeared to be lower than those of human samples. Zimmerman (1980) used static light scattering principles and the assumption that the vitreous is a spherical pendulum to calculate the shear modulus of the vitreous. This is the only paper to date that reported the *in vivo* modulus of the vitreous from patients aged 18, 26, 28, 38, 47, and 50 years old. No differences due to age were found, and the calculated elastic shear modulus was 0.05 Pa, which is at least an order of magnitude lower than other reported values. Weber et al. (1982) performed periodic oscillations of a magnetic sphere embedded in the vitreous to calculate the spring constant and damping factor of human vitreous. The age of the human donors ranged from 44 to 73 years. It is unclear how spring constant and damping factor can be related to modulus. Shafaie et al. (2018) is the only study to date that used shear rheometry to measure the rheological properties of the human vitreous. The ages of the donors were 48, 61, 70, 71, and 92. Unfortunately, rheological properties of the vitreous were not intensively investigated and reported, as the focus of this paper was the transport properties of drugs inside the vitreous. The number of human samples was also limited (5 pairs).

**Table 2:** Summaries of rheological data of the vitreous humor.

**Species**	**Paper**	**Technique**	**Sample size**	**Data type**	**Value**
Human	This study	Shear rheometry	*n* = 23	Storage modulus	G′ = 6.5 ± 3.0 Pa
				Loss modulus	G″ = 0.96 ± 0.47 Pa
	Shafaie et al., 2018	Shear rheometry	*n* = 3	Storage modulus	G′ = 1.4 ± 0.95 Pa
				Loss modulus	G″ = 0.7 ± 0.37 Pa
	Lee et al., 1992	Microrheometry	*n* = 20	Internal elastic modulus	1.2–2.5 Pa
	Weber et al., 1982	Periodic oscillations	*n* = 8	Spring constant	D_0_/r^2^π = 76,000 ± 8,200 N/m3
				Damping factor	r_z_/r^2^ = 2,940 ± 380 N*s/m
	Zimmerman, 1980	Light scattering	*n* = 6	Elastic shear modulus	0.05 Pa
Porcine	This study	Shear rheometry	*n* = 15	Storage modulus	G′ = 5.0 ± 0.58 Pa
				Loss modulus	G″ = 0.65 ± 0.22 Pa
	Shafaie et al., 2018	Shear rheometry	*n* = 3	Storage modulus	G′ = 1.4 ± 0.14 Pa
				Loss modulus	G″ = 0.4 ± 0.14 Pa
	Filas et al., 2014	Shear rheometry	*n* = 8	Storage modulus	G′ = 4–10 Pa
				Loss modulus	G″ = 1–2 Pa
	Sharif-Kashani et al., 2011	Shear rheometry	*n* = 3	Storage modulus	G′ = 1.1 ± 0.2 Pa
				Loss modulus	G″ = 0.3 ± 0.1 Pa
	Swindle-Reilly et al., 2009	Capillary rheometry	*n* = 87	Storage modulus	G′ = 0.3–8 Pa
				Loss modulus	G″ = 0.2–3 Pa
	Swindle et al., 2008	Capillary rheometry	*n* = 15	Storage modulus	G′ = 0.07–2 Pa
				Loss modulus	G″ = 0.08–0.8 Pa
				Elastic Modulus	E = 57.3 ± 5.5 Pa
	Nickerson et al., 2005, 2008	Shear rheometry	*n* = 9	Storage modulus	G′ = 2.8 ± 0.9 Pa
				Loss modulus	G″ = 0.7 ± 0.4 Pa
	Lee et al., 1994	Microrheometry	*n* = 20	Internal elastic modulus	0.8–1.0 Pa
	Shafaie et al., 2018	Shear rheometry	*n* = 3	Storage modulus	G′ = l.7 ± 0.31Pa
				Loss modulus	G″ = 0.7 ± 0.12 Pa
	Filas et al., 2014	Shear rheometry	*n* = 8	Storage modulus	G′ = 10–23 Pa
				Loss modulus	G″ = 5 Pa
	Zimberlin et al., 2010	Cavitation rheology	*n* = 5–10	Storage modulus	G′ = 660 Pa (*in vivo*)
					G′ = 120 Pa (*ex vivo*)
Bovine	Nickerson et al., 2005, 2008	Shear rheometry	*n* = 17	Storage modulus	G′ = 7.0 ± 2.0 Pa
				Loss modulus	G″ = 2.2 ± 0.6 Pa
	Lee et al., 1994	Microrheometry	*n* = 20	Internal elastic modulus	1.2–2.7 Pa
	Tokita et al., 1984	Torsion pendulum		Storage modulus	G′ = 0.l−1 Pa
				Loss modulus	G″ = 0.1–1 Pa
	Weber et al., 1982	Periodic oscillations	*n* = 8	Spring constant	D_0_/r^2^π = 60,000 ± 6,000 N/m^3^
				Damping factor	r_z_/r^2^ 2,815 ± 264 N*s/m
	Bettelheim and Wang, 1976	Compression chucks	*n* = 5	Storage modulus	G′ = 4.2–4.6 Pa
				Loss modulus	G″ = 1.9–3.6 Pa
Leporine	Silva et al., 2017	Shear rheometry	*n* = 14	Storage modulus	G′ = 1.86 ± 1.14 Pa
				Loss modulus	G″ = 0.61 ± 0.39 Pa
	Watts et al., 2014	Microrheometry	*n* = 10	Storage modulus	G′ = 0.014–0.14 Pa
				Loss modulus	G″ = 0.006–0.11 Pa
Ovine	Shafaie et al., 2018	Shear rheometry	*n* = 3	Storage modulus	G′ = 4.2 ± 0.62 Pa
				Loss modulus	G″ = 2.3 ± 0.56 Pa
	Colter et al., 2015	Shear rheometry	*n* = 30	Storage modulus	G′ = 10–170 Pa
				Loss modulus	G″ = 10–170.86 Pa
Hircine	Suri and Banerjee, 2006	Shear rheometry		Storage modulus	G′ = 1,000 Pa
				Loss modulus	G″ = 400 Pa

Until now, no age-related changes in the mechanical properties of the human vitreous have been reported. Although the volume of liquid vitreous is well-known to increase with age due to phase separation, quantitative measurements of age-related vitreous liquefaction remain elusive, especially in terms of mechanical properties. This study demonstrated, for the first time, that the vitreous gel becomes stiffer while the vitreous liquid becomes less elastic with age. This was validated over a range of physiologically relevant frequencies. Specifically, for the vitreous samples from donors aged more than 65 years old, their moduli and viscosity are significantly larger than those of the young vitreous samples aged <65 years old and those of porcine vitreous (Figure 6, ${\text{G}}_{\text{old human}}^{*}$ = 1.11 ± 0.64 Pa > ${\text{G}}_{\text{young human}}^{*}$ = 0.53 ± 0.39 Pa and ${\text{G}}_{\text{porcine}}^{*}$ = 0.24 ± 0.16 Pa, *p* < 0.0001). Similar results were found when the age differentiation was changed from 65 years old to either 50 years old, which separates the two very young samples (20 and 24 years old, *p* = 0.002), or 57 years old (*p* = 0.002), which is the average age of patients receiving vitrectomy (Wubben et al., 2016).

The main goal of this paper was to determine the correlation between age and rheological properties of the human vitreous. The biochemical compositions of the human, porcine, bovine, and ovine vitreous have been previously reported (Shafaie et al., 2018), along with the correlation between age and biochemical compositions of the human vitreous (Sebag, 1987). The vitreous composition-biomechanical relationship has also been studied using enzymatic digestion (Filas et al., 2014). In this study, the solid part of the old human vitreous humor was found to be stiffer than the porcine vitreous humor (Figure 6). These findings are consistent with the data presented in Lee et al. (1992, 1994), in which the internal elastic modulus of the porcine vitreous was found to be slightly lower than that of the human vitreous. The collagen concentration in human vitreous (280–1,360 μg/ml) is higher than that in the porcine vitreous (150 μg/ml), while the hyaluronic acid concentration is similar between the species (human: 140–340 μg/ml; porcine: 160 μg/ml) (Shafaie et al., 2018). Since collagen is one of the components that contributes to the stiffness of the vitreous gel (Filas et al., 2014), it is reasonable to speculate that the old human vitreous humor is stiffer than the porcine vitreous humor due to the higher concentration of collagen in the human vitreous. The differences in the parameters of the discrete retardation model between this study and Sharif-Kashani et al. (2011) further corroborate this idea. The first retardation time and compliance, which reflects the collagen response, are significantly different between human and pig (Table 1). This difference could, again, be due to the difference in the concentration of collagen between human and porcine vitreous. However, no differences were found in the second retardation time and compliance (hyaluronic acid response) between the human and porcine vitreous, mirroring the similarity in the concentration of hyaluronic acid in the human and porcine vitreous.

During testing, areas of stiffened vitreous gel in some of the older vitreous samples were observed. This finding can be counter-intuitive, since vitreous liquefaction implies that the vitreous becomes more liquid-like and softens with age. However, it has been shown that the concentration of hyaluronic acid decreases while the concentration of collagen increases in the vitreous gel as one ages (Sebag, 1987). Nickerson et al. (2008) proposed a hypothesis that hyaluronic acid provides a swelling effect on the hyaluronic acid-collagen network and can be readily expulsed out of the network in certain circumstances. Filas et al. (2014) suggested that excessive water loss in the vitreous (potentially due to the expulsion of hyaluronic acid) could increase the stiffness of the gel due to the collapse of structural proteins (e.g., collagen) beyond a critical density. Silva et al. (2017) found that the solid phase of rabbit vitreous was stiffer 4 h post-dissection compared to the gel phase immediately after dissection. Although not discussed by the authors, the increase in stiffness of samples after dissection could be due to the expulsion of hyaluronic acid from the vitreous gel. The gel phase, with a decreased concentration of hyaluronic acid, might stiffen due to dehydration. Filas et al. (2014) found that vitreous digested with hyaluronidase has a lower loss tangent, meaning the vitreous is more elastic-like compared to vitreous digested with collagenase. The same mechanism might occur *in vivo* in the eye. Phase separation of the vitreous gel due to aging is hypothesized to create pockets of liquid rich in hyaluronic acid. Expulsion of hyaluronic acid causes the vitreous gel to become dehydrated and artificially stiffened due to decreased water content. Nickerson et al. (2008) showed a rapid liquid expulsion within 5 min of dissection, resulting in a decrease in weight of the vitreous humor. Vitreous liquefaction might therefore be characterized as an increase of liquid volume in the vitreal chamber due to expulsion of hyaluronic acid from the collagen-hyaluronic acid gel network and stiffening of the vitreous gel.

The degradation of collagen type IX was shown to be one of the causes of vitreous liquefaction (Le Goff, 2008). Collagen type IX normally coats the outer surface of collagen type II in the vitreous, preventing the collagen fibrils from adhering to each other. Collagen type IX was found to have a half-life of 11 years, with an exponential decrease in concentration in human vitreous starting at a young age. With increasing age, more collagen type IX is lost on the surface of the vitreous collagen fibrils, leading to an increased possibility that the fibrils aggregate. The aggregation of collagen fibrils results in the collapse of the vitreous and expulsion of the hyaluronic acid inside the collagen-hyaluronic acid network. The collapse of the collagen network increases its degree of crosslinking, which might be the reason for the detected stiffening of the gel portion of the vitreous with age. Filas et al. (2014) showed that the vitreous gel is completely liquefied with total collagen degradation using collagenase. This observation resembles the formation of the liquid phase of the vitreous liquid with aging due to hyaluronic acid expulsion from the collapsed collagen network.

While the degradation of vitreous humor causes the once homogeneous gel to separate into the heterogeneous phases, the evolution of solid and liquid vitreous components with frequency is similar (Figure 4). The modulus increases and the viscosity decreases with frequency for both the solid and liquid phases. The storage modulus of the liquid phase is higher than its loss modulus for the range of frequencies tested. This suggests that, from a rheological standpoint, the liquid phase still behaves as a “gel.” Silva et al. (2017) also found a similar response in the liquid phase of the rabbit vitreous humor. This observation might explain the similarities in the modulus and viscosity response to frequency between the liquid and solid phases. The *in vivo* vitreous connects to specific locations inside the eye, giving a complex mechanical response that not only depends on the material properties of the vitreous humor but also on the properties of the corneoscleral shell and other appendages. The heterogenized vitreous humor can disrupt this stress distribution balance in the eye, potentially putting more stress on the retina which could cause retinal detachment or macular holes. This is an interesting biomechanical problem that could be investigated in future studies.

Colter et al. (2015) tested whole ovine vitreous with the hyaloid membrane and found that the dynamic moduli of the adult vitreous was about 2 times lower than infant vitreous, albeit not statistically significant. While this observation appears contradictory with the findings of this study, it should be noted that the entire vitreous was tested in their study. The adult vitreous specimens in their study likely had pockets of liquid that, when tested as a whole with the vitreous gel, might lower the dynamic moduli compared to those from the infant ovine vitreous, which would have less liquid phase in the vitreous. In other words, although the adult vitreous appeared to be macroscopically softer in their study, it might have been due to the increase of liquid volume due to age. This study further investigated this phenomena by testing the liquid and solid phases of the vitreous separately and found that the gel portion of the vitreous stiffens with age, similar to many other tissues in the body.

It should be noted that due to the scarcity of young human donors, the donor age was not normally distributed. As such, the human data was only divided to two somewhat crude categories as “young” and “old.” Collecting more samples from donors aged 50 or below will allow for greater resolution of changes to mechanical properties of the vitreous humor at different stages of development. These data showed a qualitative exponential relationship. While this was used to demonstrate the correlation between age and modulus/viscosity (Figure 5B), more data points for younger samples are needed to accurately determine these correlations. The uneven spread of male and female specimens also made it difficult to discern any significant correlations based on sex. Additionally, the human eye samples were used for other research purposes before the vitreous humor could be harvested, causing an extended post-mortem time before the vitreous humor could be tested. However, the porcine post-mortem and post-extraction studies demonstrated no statistical differences in rheological properties of the porcine vitreous found for samples obtained within 48 h post mortem and used within 4 h post-dissection, compared to fresh samples. Rheological properties of vitreous samples were also previously found to not be affected by storage as intact globes for up to 60 h post-mortem (Weber et al., 1982).

This study marks the first investigation of age-related changes to the mechanical properties of human vitreous using shear rheology. Dynamic moduli and viscosity of the old human vitreous humor were found to be larger than those of younger human and porcine vitreous humor. The stiffness of the gel portion of the vitreous humor was found to be positively correlated with age. This counterintuitive stiffening of the solid component of the vitreous with age could be explained based on the dehydration of the vitreous gel due to the expulsion of hyaluronic acid and increase in collagen concentration. Vitreous liquefaction might therefore consist of both an increase in volume of liquid vitreous and a stiffening of the shrinking vitreous gel due to dehydration. The simultaneous and localized liquefaction and stiffening of the vitreous humor can alter the stress distribution of the surrounding tissues, potentially causing vitreous-related complications such as retinal tears and macular holes. A better understanding of the rheological properties of the vitreous humor and its age-related changes will allow for better biomimetic vitreous substitutes, enhanced transport of therapeutic drugs in the vitreous, and improved diagnosis of vitreoretinal diseases.

## Author Contributions

All authors were involved in the conception and design of the study. NT collected all of the data, performed the statistical analysis, and wrote the initial draft of the manuscript. KS-R critically reviewed and assisted in the preparation of the manuscript. All authors were involved in the interpretation of the data, made critical contributions to the manuscript, approved the final version of the manuscript, and take responsibility for the findings of the study.

### Conflict of Interest Statement

The authors declare that the research was conducted in the absence of any commercial or financial relationships that could be construed as a potential conflict of interest.
