# Erratum: Rheological Properties and Age-Related Changes of the Human Vitreous Humor

**DOI:** 10.3389/fbioe.2019.00044

**Published:** 2019-03-15

**Authors:** Frontiers Production Office

**Affiliations:** Frontiers Production Office, Frontiers Media SA, Lausanne, Switzerland

**Keywords:** rheometry, vitreous, aging, ocular biomechanics, liquefaction, eye, viscoelasticity, floaters

Due to a production error, the value of the Loss Modulus Data Type, for the Human species in the last column of Table 2, was erroneously changed.

**Table 2:** Summaries of rheological data of the vitreous humor.

**Species**	**Paper**	**Technique**	**Sample size**	**Data type**	**Value**
Human	This study	Shear rheometry	*n* = 23	Storage modulus	G′ = 6.5 ± 3.0 Pa
				Loss modulus	G″ = 0.96 ± 0.47 Pa
	Shafaie et al., 2018	Shear rheometry	*n* = 3	Storage modulus	G′ = 1.4 ± 0.95 Pa
				Loss modulus	G″ = 0.7 ± 0.37 Pa
	Lee et al., 1992	Microrheometry	*n* = 20	Internal elastic modulus	1.2–2.5 Pa
	Weber et al., 1982	Periodic oscillations	*n* = 8	Spring constant	D_0_/r^2^π = 76,000 ± 8,200 N/m3
				Damping factor	r_z_/r^2^ = 2,940 ± 380 N*s/m
	Zimmerman, 1980	Light scattering	*n* = 6	Elastic shear modulus	0.05 Pa
Porcine	This study	Shear rheometry	*n* = 15	Storage modulus	G′ = 5.0 ± 0.58 Pa
				Loss modulus	G″ = 0.65 ± 0.22 Pa
	Shafaie et al., 2018	Shear rheometry	*n* = 3	Storage modulus	G′ = 1.4 ± 0.14 Pa
				Loss modulus	G″ = 0.4 ± 0.14 Pa
	Filas et al., 2014	Shear rheometry	*n* = 8	Storage modulus	G′ = 4–10 Pa
				Loss modulus	G″ = 1–2 Pa
	Sharif-Kashani et al., 2011	Shear rheometry	*n* = 3	Storage modulus	G′ = 1.1 ± 0.2 Pa
				Loss modulus	G″ = 0.3 ± 0.1 Pa
	Swindle-Reilly et al., 2009	Capillary rheometry	*n* = 87	Storage modulus	G′ = 0.3–8 Pa
				Loss modulus	G″ = 0.2–3 Pa
	Swindle et al., 2008	Capillary rheometry	*n* = 15	Storage modulus	G′ = 0.07–2 Pa
				Loss modulus	G″ = 0.08–0.8 Pa
				Elastic Modulus	E = 57.3 ± 5.5 Pa
	Nickerson et al., 2005, 2008	Shear rheometry	*n* = 9	Storage modulus	G′ = 2.8 ± 0.9 Pa
				Loss modulus	G″ = 0.7 ± 0.4 Pa
	Lee et al., 1994	Microrheometry	*n* = 20	Internal elastic modulus	0.8–1.0 Pa
	Shafaie et al., 2018	Shear rheometry	*n* = 3	Storage modulus	G′ = l.7 ± 0.31Pa
				Loss modulus	G″ = 0.7 ± 0.12 Pa
	Filas et al., 2014	Shear rheometry	*n* = 8	Storage modulus	G′ = 10–23 Pa
				Loss modulus	G″ = 5 Pa
	Zimberlin et al., 2010	Cavitation rheology	*n* = 5–10	Storage modulus	G′ = 660 Pa (*in vivo*)
					G′ = 120 Pa (*ex vivo*)
Bovine	Nickerson et al., 2005, 2008	Shear rheometry	*n* = 17	Storage modulus	G′ = 7.0 ± 2.0 Pa
				Loss modulus	G″ = 2.2 ± 0.6 Pa
	Lee et al., 1994	Microrheometry	*n* = 20	Internal elastic modulus	1.2–2.7 Pa
	Tokita et al., 1984	Torsion pendulum		Storage modulus	G′ = 0.l−1 Pa
				Loss modulus	G″ = 0.1–1 Pa
	Weber et al., 1982	Periodic oscillations	*n* = 8	Spring constant	D_0_/r^2^π = 60,000 ± 6,000 N/m^3^
				Damping factor	r_z_/r^2^ 2,815 ± 264 N*s/m
	Bettelheim and Wang, 1976	Compression chucks	*n* = 5	Storage modulus	G′ = 4.2–4.6 Pa
				Loss modulus	G″ = 1.9–3.6 Pa
Leporine	Silva et al., 2017	Shear rheometry	*n* = 14	Storage modulus	G′ = 1.86 ± 1.14 Pa
				Loss modulus	G″ = 0.61 ± 0.39 Pa
	Watts et al., 2014	Microrheometry	*n* = 10	Storage modulus	G′ = 0.014–0.14 Pa
				Loss modulus	G″ = 0.006–0.11 Pa
Ovine	Shafaie et al., 2018	Shear rheometry	*n* = 3	Storage modulus	G′ = 4.2 ± 0.62 Pa
				Loss modulus	G″ = 2.3 ± 0.56 Pa
	Colter et al., 2015	Shear rheometry	*n* = 30	Storage modulus	G′ = 10–170 Pa
				Loss modulus	G″ = 10–170.86 Pa
Hircine	Suri and Banerjee, 2006	Shear rheometry		Storage modulus	G′ = 1,000 Pa
				Loss modulus	G″ = 400 Pa

The publisher apologizes for this mistake. The original article has been updated.
