# Supplementary figures and images for: Rheological Properties and Age-Related Changes of the Human Vitreous Humor
Source: Front Bioeng Biotechnol. 2018 Dec 18;6:199. doi: 10.3389/fbioe.2018.00199 (PMC6305337; doi:10.3389/fbioe.2018.00199)

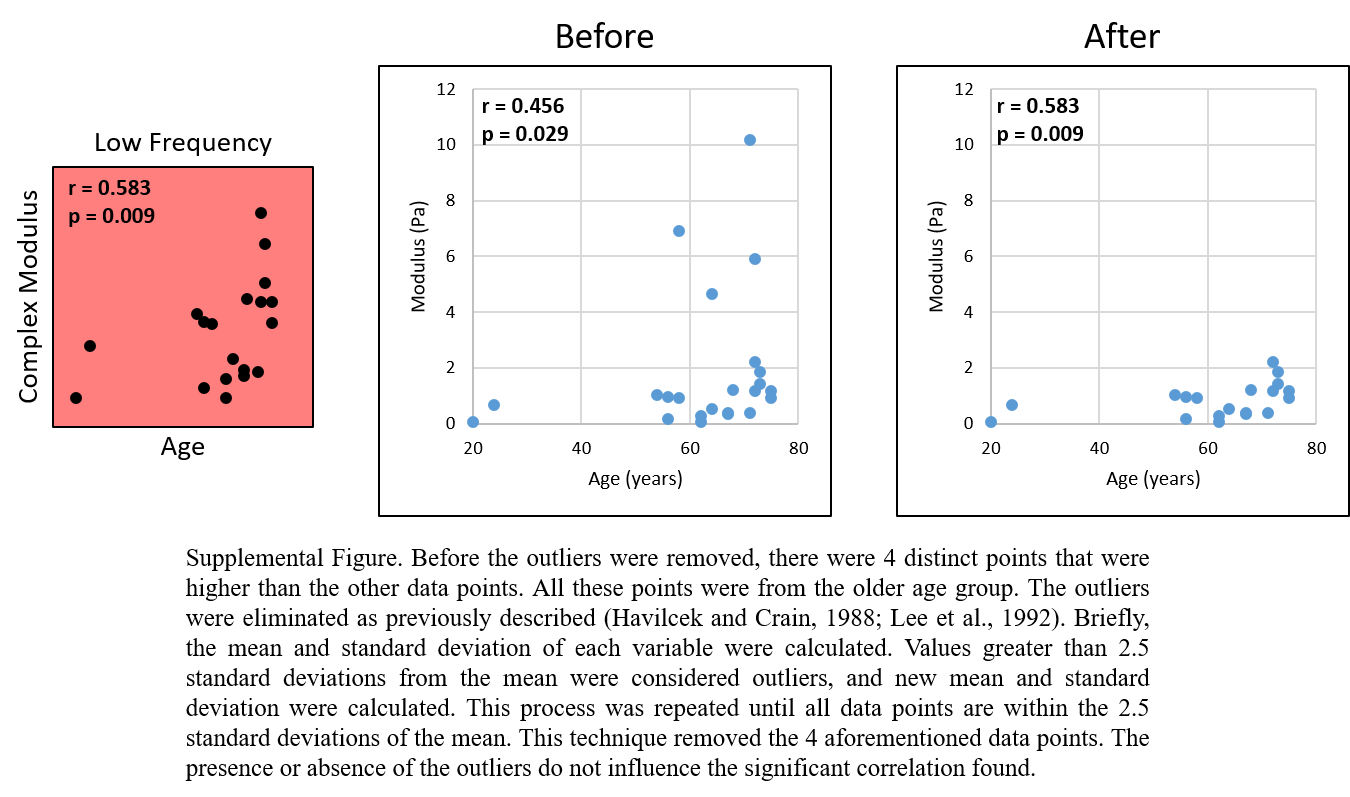

Supplement: Supplementary file 1 [file Image_1.TIF]
